# Read-depth based approach on whole genome resequencing data reveals important insights into the copy number variation (CNV) map of major global buffalo breeds

**DOI:** 10.1186/s12864-023-09720-8

**Published:** 2023-10-16

**Authors:** Sheikh Firdous Ahmad, Celus Chandrababu Shailaja, Sakshi Vaishnav, Amit Kumar, Gyanendra Kumar Gaur, Sarath Chandra Janga, Syed Mudasir Ahmad, Waseem Akram Malla, Triveni Dutt

**Affiliations:** 1 Division of Animal Genetics, ICAR-Indian Veterinary Research Institute, Izatnagar, Bareilly, Uttar Pradesh 243122 India; 2 Luddy School of Informatics, Computing & Engineering, Indiana University Indianapolis (IUI), Indianapolis, 46202 USA; 3 Division of Animal Biotechnology, Faculty of Veterinary Sciences and AH, Sher-e-Kashmir University of Agricultural Sciences and Technology, Srinagar, Jammu & Kashmir 190006 India; 4 Division of Veterinary Biotechnology, ICAR-Indian Veterinary Research Institute, Izatnagar, Bareilly, Uttar Pradesh 243122 India

**Keywords:** Buffalo, CNV, CNVR, Evolution, Read depth, WGS

## Abstract

**Background:**

Elucidating genome-wide structural variants including copy number variations (CNVs) have gained increased significance in recent times owing to their contribution to genetic diversity and association with important pathophysiological states. The present study aimed to elucidate the high-resolution CNV map of six different global buffalo breeds using whole genome resequencing data at two coverages (10X and 30X). Post-quality control, the sequence reads were aligned to the latest draft release of the Bubaline genome. The genome-wide CNVs were elucidated using a read-depth approach in CNVnator with different bin sizes. Adjacent CNVs were concatenated into copy number variation regions (CNVRs) in different breeds and their genomic coverage was elucidated.

**Results:**

Overall, the average size of CNVR was lower at 30X coverage, providing finer details. Most of the CNVRs were either deletion or duplication type while the occurrence of mixed events was lesser in number on a comparative basis in all breeds. The average CNVR size was lower at 30X coverage (0.201 Mb) as compared to 10X (0.013 Mb) with the finest variants in Banni buffaloes. The maximum number of CNVs was observed in Murrah (2627) and Pandharpuri (25,688) at 10X and 30X coverages, respectively. Whereas the minimum number of CNVs were scored in Surti at both coverages (2092 and 17,373). On the other hand, the highest and lowest number of CNVRs were scored in Jaffarabadi (833 and 10,179 events) and Surti (783 and 7553 events) at both coverages. Deletion events overnumbered duplications in all breeds at both coverages. Gene profiling of common overlapped genes and longest CNVRs provided important insights into the evolutionary history of these breeds and indicate the genomic regions under selection in respective breeds.

**Conclusion:**

The present study is the first of its kind to elucidate the high-resolution CNV map in major buffalo populations using a read-depth approach on whole genome resequencing data. The results revealed important insights into the divergence of major global buffalo breeds along the evolutionary timescale.

**Supplementary Information:**

The online version contains supplementary material available at 10.1186/s12864-023-09720-8.

## Background

Livestock contributes significantly to the national gross domestic product (GDP) of developing countries, including India. India is home to a large inventory of multiple farm animal species including more than 300 million bovines i.e., cattle and buffaloes. Buffaloes contribute significantly to the national GDP of India and its milk production and farmers’ profitability. It ensures the nutritional security of the masses in developing countries. More than half of the global bubaline population is reared in India with two main subspecies, riverine (*Bubalus bubalis bubalis*) and the swamp (*Bubalus bubalis carabanensis*). The two sub-species of buffaloes are characterized by distinct morphology and karyotype characteristics [1, 2]. Globally, the buffalo population represents 208 million heads [3]. India is the hotspot for buffalo biodiversity, mirrored by 20 recognized buffalo breeds [4]. Various Indian buffalo breeds, with improved genetic merit and performance vis-à-vis economic traits, have reached transboundary distribution across various global nations. Murrah, widely known as ‘Black gold’, is a transboundary milch breed of buffaloes accounting for 42.8% of the total Indian buffalo population. It has high milk production potential and distribution across multiple nations. Bhadawari buffaloes produce milk with high-fat content, which can range up to 13%. Jaffarabadi is one of the best milking riverine breeds, well known for its higher milk fat content and heavy body suitable for draught purposes. Banni buffaloes, believed to have evolved by the efforts of the local ‘Maldhari’ community of Gujarat, possess a unique gene pool that allows it to thrive in harsh climatic conditions. On the other hand, Surti and Pandharpuri are medium-sized breeds that can be distinguished by their sickle and sword-shaped horns, respectively. Multiple populations of these buffalo breeds have been imported by different global nations for improvement of their animal genetic resource (AnGR) base [5].

Buffaloes have evolved through more than 5000 years of domestication, leading to the adoption of morpho-physiological, and adaptive features useful in harsh tropical and humid environments. Buffaloes are ideally considered the future animals of choice to meet the ever-growing animal protein demand [6]. Buffaloes contribute around 45% to India’s total milk production. Buffalo milk is nutritionally rich with high-fat content and essential minerals and is thus recommended to produce cheese, yogurt, and cream. Besides the meat (carabeef), they also provide horns and hides, though their maximum export potential is still untapped.

Globally, numerous studies have focused on the genetic analyses of economic traits and genetic diversity in buffaloes [7, 8]. Various molecular markers, especially microsatellites and single nucleotide polymorphism (SNP) have gained increased penetrance into modern animal breeding programs wherein different structural and functional genetic variants are used to select animals for improved performance in future generations [9]. Previously, researchers utilized microsatellite markers to analyze the divergence time between swamp and river buffalo and succeeded in confirming their distinct genetic origins [10]. However, the introduction of genome assemblies and SNP chips has facilitated studies at the genome level, enabling the detection of QTLs associated with performance traits and variant detection [11, 12]. The successful release of the first haplotype-phased reference genome assembly, NDDB_SH_1, for the riverine buffalo has been a significant milestone in buffalo genomics. Along with the emergence of second and third-generation sequencing platforms, whole genome resequencing (WGS) has become more accessible, allowing for the discovery of genetic variants (structural and functional) and molecular markers with higher accuracy. It is coupled with increased and easy accessibility to bioinformatics and statistical programs that are useful to analyze the WGS data in livestock species.

Copy number variations, as structural variants, have gained significance with respect to their inheritance and association with multiple traits of economic interest and pathophysiological states. Copy number variants are unbalanced structural variants, conventionally defined as the fragments of the genome with sizes ranging from a few kilobases to 5 megabases [13]. CNVs are distinct from SNPs and Indels and occupy larger genomic spaces than other variants. In humans, 4.8–9.5% of the genome consists of CNVs, unlike the contribution from SNPs, which comes to only around 0.1% [14]. Consequently, they alter the gene dosage and genomic regulation, or cause position effects [15], resulting in drastic changes in gene expression. Different approaches have been used to elucidate the copy number variations at the genome-wide level in livestock. Initially, lower-resolution comparative genomic hybridization arrays were used, but PCR-based methods improved the resolution during subsequent times. Currently, array genotyping data and whole genome/exome/amplicon sequencing data are routinely studied while using various algorithms including PennCNV [16], CNVnator [17], CNVcaller [18], cn.MOPS [19] and others.

Various studies have reported the elucidation of copy number variations at a genome-wide scale in farm animal species including cattle [20], buffaloes [21], equine [22], pigs [23], yak [24], and chicken [25]. Furthermore, the association of CNVs with traits of economic interest has also been reported in livestock populations [9, 26, 27]. Keeping in view the aforementioned points, the present was undertaken to elucidate the CNV maps in six breeds of Indian riverine buffaloes using WGS data at two coverages. Breed-differentiated CNVs were detected, and genes overlapping the CNVs were also identified.

## Results and discussion

### Descriptive statistics

**Fig. 1:**
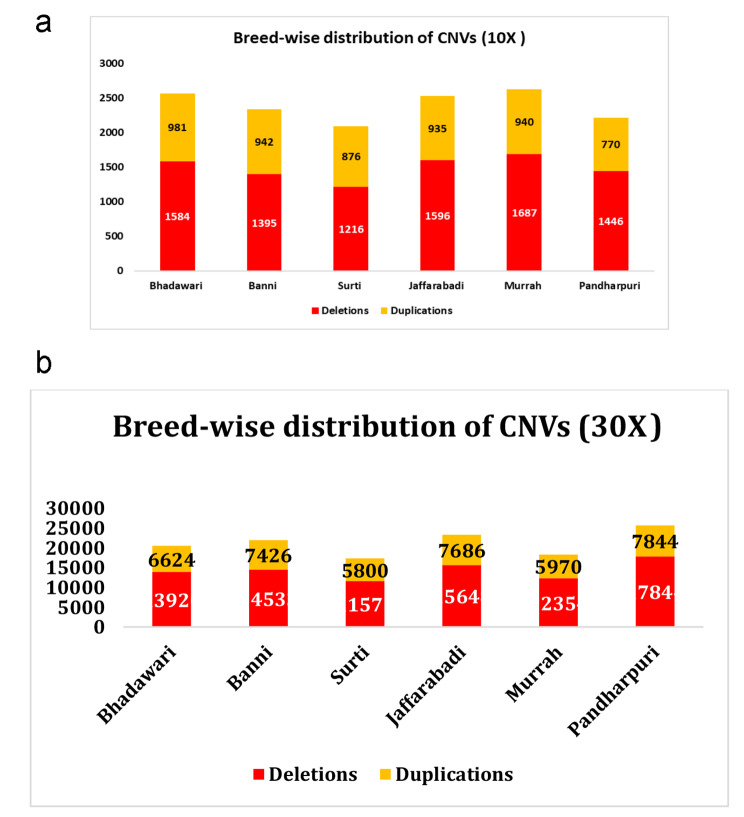
(**a**): CNV distribution of six buffalo breeds using a read-depth based approach on whole genome resequencing data at 10X coverage. (**b**). CNV distribution of six buffalo breeds using a read-depth based approach on whole genome resequencing data at 30X coverage

The latest advancements in whole genome resequencing enable the accurate detection of both common and rare CNVs. It enables the identification of smaller and naive (previously unknown ones) genetic variants down to the level of individual base pairs. The whole genome resequencing approach presents many benefits for the elucidation of structural variants including CNVs. The read depth-based methods are recommended for Illumina NGS data [15] as they do not require the reference sample and express the exact CNV counts rather than their positions. This method is adopted in CNVnator, which uses a mean shift-based (MSB) approach for accurate variant calling [28], better sensitivity and a low false-discovery rate [17]. In this study, WGS data on multiple buffalo breeds were used to elucidate the CNV and CNVR maps using a read-depth-based approach in CNVnator. Bin sizes of 1000 and 100 were found to be optimal to assess the CNVs in different buffalo breeds at 10X and 30X coverages, respectively. The read-depth approach in CNVnator is based on binning read-depth signals which are dependent on sequencing coverage and read length [29]. Therefore, an integrated run of all the samples could not be attempted in this study. However, instead of merging the twin coverage datasets, an opportunity was explored to present the CNV maps at both these coverages and report CNV/ CNVR maps in these global buffalo breeds with separate downstream processing. Post-filtering, 14,368 CNVs (8924 deletions and 5444 duplications) were scored at 10X coverage covering six global buffalo breeds. The maximum number of CNVs was observed in Murrah (2627) while Surti scored the minimum number (2092). Yang et al. [30] found 21,152 CNV regions in a whole genome dataset of 20 buffalo breeds comprising 141 buffaloes using LUMPY and CNVnator. Strillacci et al. [31] elucidated the CNV profile of Iranian river buffaloes using 90K genotyping array data and reported lesser number of structural variants (9550 CNVs, representing 1.97% of the buffalo genome) with a loss-gain ratio of more than one. The inconsistency in CNV counts within the same species is attributable to differences in CNV detection algorithms; sample size; the evolutionary history of the population/ breed; and data used for calling the structural variants. Additionally, the elucidation of structural variants including CNVs from NGS data is greatly influenced by other factors such as read length, sequence coverage, GC bias and mappability of next-generation sequencing platforms [32]. The number and distribution of CNVs were elucidated at two coverages at whole genome level i.e., 10X and 30X. The breed-wise descriptive statistics of CNVs have been illustrated in Fig. 1(a) and Fig. 1(b).

On the other hand, 1,27,222 CNVs were obtained across all the breeds at 30X coverage. Similar estimates have been reported in previous studies using WGS data in other species including 1,82,823 CNVs in cattle [33], 2,08,649 CNVs in goats [34] and 1,64,733 CNVs in mink [35]. Interestingly, the maximum number of total CNVs was found in Pandharpuri (25,688); however, a minimum number of CNVs were scored in Surti (17,373), consistent with the results at lower (10X) coverage. The differences in the number of CNVs explain the genetic variability between the species or breeds, especially in terms of their evolutionary history, effective population size and other similar attributes [36]. In terms of overlapping (at least 1 bp overlap) and unique (no overlap) occurrences of CNVs within and across breeds (Table 1), Pandharpuri had the maximum number with 43,788 hits (10X versus 30X comparison), while Murrah had the highest count for unique with 335 hits (10X versus 30X comparison).

**Table 1:** Summary of overlapping and unique hits of CNVs within and across breeds at twin coverages

Breeds	Banni	Bhadawari	Pandharpuri	Murrah	Surti	Jaffarabadi
**Banni**	*25,562* *(197)*	10,850 *(218)*	9359 *(229)*	11,194 *(181)*	8790 *(201)*	10,759 *(190)*
**Bhadawari**	68,628 *(4784)*	*29,981* *(54)*	10,673 *(237)*	12,649 *(153)*	9504 *(227)*	11,931 *(214)*
**Pandharpuri**	68,410 *(5131)*	80,000 *(2901)*	*43,788* *(38)*	11,045 *(142)*	8242 *(181)*	10,503 *(146)*
**Murrah**	62,108 *(4923)*	65,799 *(3508)*	65,412 *(8260)*	*23,800* *(335)*	9924 *(214)*	12,361 *(181)*
**Surti**	60,696 *(5481)*	64,399 *(3925)*	63,822 *(8942)*	58,103 *(3153)*	*19,964* *(217)*	9432 *(179)*
**Jaffarabadi**	73,238 *(3388)*	78,012 *(2402)*	90,399 *(3495)*	67,950 *(2421)*	67,009 *(1887)*	*36,096* *(44)*

Diagonal elements refer to overlap between 10X and 30X of the same breed. The upper triangle represents the overlap between two breeds at 10X coverage. The lower triangle indicates overlap between two breeds at 30X coverage. The italicized values in parenthesis represent the unique hits with no overlap across specific comparisons.

The proportion of CNV types was also estimated besides the assessment of the CNV counts. In each of the breeds assessed at both the coverages, deletion events overnumbered the duplications, as depicted in Fig. 1(a) and Fig. 1(b). The finding was concurrent with previous NGS studies reported in cattle [33] and buffaloes [30]. Moreover, the deletion events were abundantly reported from aCGH arrays due to ascertainment bias, pinpointing that deletions are effectively captured by them as compared to the other analytical methods [37]. In addition, Turner et al. [38] have reported that non-allelic homologous recombination (NAHR), which is one of the potential mechanisms of CNV generation, is more likely to result in deletions as compared to duplications. However, an opposite trend has also been reported in horse populations with duplications exceeding the deletion events [22, 39].

In this study, the percentage of loss and gain events were estimated separately for all six breeds, among which Pandharpuri scored the maximum percentage of deletions at both coverages (65.25% and 69.46% at 10X and 30X, respectively). On the other hand, Surti showed the highest percentage of duplications (41.87%) at 10X while Banni scored the highest proportion estimate (33.82%) at 30X coverage. The breeds under study showed a loss/gain ratio ranging from 1.39 to 1.88 at lower sequencing coverage. When the coverage was increased to 30X, the ratio varied from 1.96 to 2.27, with Pandharpuri having the highest ratio at both the coverages. Similar estimates have been reported by Strillacci et al. [31] in Iranian buffaloes, where the Mazandarani breed had the highest ratio of 1.32. However, in the polled yak, Jia et al. [24] reported a very high ratio of loss to gain events (15.56:1) using Bovine HD bead chip genotyping data for CNV detection. The differences in loss and gain events inferred that there was a net loss of genetic material in all the breeds being studied.

The disparity in CNV length is also evident from different studies. Considering the 10X coverage, the size varied from 5 kb to a maximum of 4.9 Mb. Whereas the minimum length of CNVs in the present study was 1.1 kb for 30X, which was close to the cut-off set for filtering. This also indicated that at higher coverage, the breakpoint resolution is more, yielding comparatively smaller segments with higher accuracy [40, 41]. The largest CNV length that was documented exceeded those found in cattle: 28 kb [42] and 129.9 kb [43]. Nonetheless, other researchers have reported maximum sizes in the Mb range in chicken, horses, and buffaloes that were in line with the above findings. For instance, in chicken, the maximum size was 4.3 Mb [44], it was 4.55 Mb in horses [22], and the longest CNV of 3.48 Mb size has been reported in buffaloes [31].

### Detection of CNVRs

**Fig. 2:**
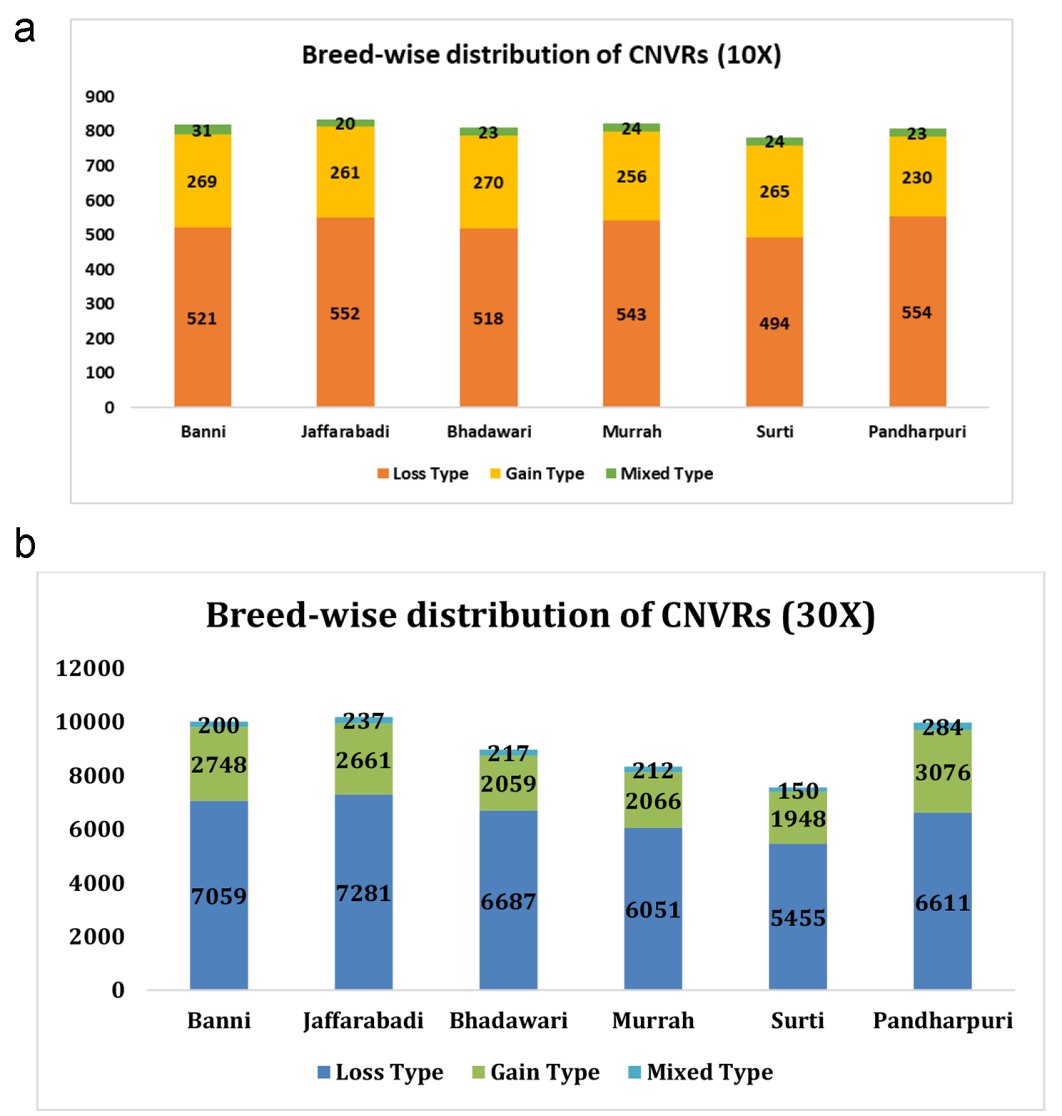
(**a**): The distribution pattern of CNVRs across six buffalo breeds on the concatenation of overlapping CNVs at 10X coverage. (**b**): The distribution pattern of CNVRs across six buffalo breeds on the concatenation of overlapping CNVs at 30X coverage

The CNVR diversity among buffalo breeds, as elucidated in the present study, has been presented in Fig. 2(a) and Fig. 2(b). At 10X coverage, the total number of CNVRs across all the breeds was 4878 with estimates ranging from 783 (Surti) to 833 (Jaffarabadi). A systematic investigation of CNVs by Liu et al. [21] identified only 1344 CNVRs in 14 water buffaloes using the read depth-based approach.

In the present study, a total of 55,002 CNVRs were recorded across all breeds at 30X coverage. The highest number of CNVRs was scored in Jaffarabadi (10,179) while the estimate was lowest in Surti (7553). Kumar et al. [45] found that despite the proximity of their breeding tracts, Jaffarabadi and Surti exhibit genetic distinctness, which may be indicated by their different CNVR count. Prior studies have also suggested that variation in CNVRs might have facilitated rapid adaptation during the domestication process and expansion of the population [46].

The CNVRs, elucidated in the present study, were classified into three categories; deletion, duplication and mixed (containing both deletions and duplications). A total of 3182, 1551, and 145 CNVRs with deletion, duplication and mixed events were scored at 10X coverage. On breed-wise examination, Pandharpuri (554) and Jaffarabadi (552) showed to have the most deletion-based CNVRs at 10X coverage. On the other hand, Bhadawari showed the highest number of duplications (270), while Banni obtained the highest count in mixed events (31). In another study, Zhang et al. [47] studied the nature of CNVRs in water buffaloes (n = 106), and recorded similar counts, with 2245 loss, 1289 gain, and 200 mixed events out of total 3734 CNV regions. It equated to only 0.88% of the reference genome assembly of Mediterranean riverine buffalo (UOA_WB_1).

The number of CNVRs within each type increased almost 10 times when the coverage was changed from 10X to 30X (3182 versus 39,144 deletion types, 1551 versus 14,558 duplication types, and 145 versus 1300 mixed types at 10X and 30X, respectively). Among different breeds, the maximum number of deletions, and duplications were observed in Jaffarabadi (7281) and Pandharpuri (3076), respectively. The latter also had the highest score for mixed-type CNVRs. Intriguingly, Surti exhibited the least counts for all three CNVR types. The average loss-gain ratio observed across all the breeds was 2.06 at 10X (varying from 1.86 to 2.41). On the other hand, the ratio was found to be higher at 30X (2.74), ranging between 2.15 and 3.25.

The unique and overlapping CNVRs were analyzed for all the breeds, and the data have been summarised in Table 2. Jaffarabadi had the highest number of overlapping hits on within-breed 10X versus 30X comparison, while Surti showed the lowest estimate. Importantly, the highest number of overlapping CNVRs were shared between Jaffarabadi and Pandharpuri, which might be indicative of possible genetic relatedness. In concurrence with the above findings, Kumar et al. [45] reported that Jaffarabadi and Pandharpuri belonged to the same lineage of buffalo breeds. Besides, the greatest fraction of overlapping CNVRs were documented for Bhadawari with 31.6% similarity between the coverages. In terms of unique hits, Murrah scored the highest (n = 114), while Pandharpuri (n = 31) showed the lowest hits.

**Table 2:** Summary of overlapping and unique hits of CNVRs within and across breeds at twin coverages

Breeds	Banni	Bhadawari	Pandharpuri	Murrah	Surti	Jaffarabadi
**Banni**	*2400* *(91)*	809 *(171)*	821 *(181)*	809 *(152)*	798 *(165)*	840 *(151)*
**Bhadawari**	7345 *(3404)*	*2832* *(39)*	805 *(163)*	829 *(121)*	784 *(167)*	813 *(153)*
**Pandharpuri**	7281 *(3516)*	8005 *(2187)*	*2922* *(31)*	829 *(122)*	793 *(146)*	841 *(121)*
**Murrah**	7306 *(3422)*	6920 *(2803)*	6893 *(3946)*	*2148* *(114)*	793 *(178)*	838 *(157)*
**Surti**	6872 *(3805)*	6560 *(3090)*	6454 *(4293)*	6540 *(2503)*	*1942* *(100)*	815 *(149)*
**Jaffarabadi**	8241 *(2674)*	8417 *(1858)*	8536 *(2800)*	7240 *(1970)*	6940 *(1474)*	*3206* *(37)*

Diagonal elements refer to overlap between 10X and 30X of the same breed. The upper triangle represents the overlap between two breeds at 10X coverage. The lower triangle indicates overlap between two breeds at 30X coverage. The italicized values in parenthesis represent the unique hits with no overlap across specific comparisons.

Table 3 presents additional information on the average size of CNVRs, genomic coverage and the perfect overlap in CNVRs (%) at two levels of sequencing coverage. The average length of CNVRs indicated that longer segments (~ 0.2 Mb) were detected at 10X while a wide variation in size (6 kb − 24.4 kb) was noted at 30X. Furthermore, the proportion of CNVR size with respect to the reference genome (2.77Gb) suggested that Murrah (6.00% at 10X) and Pandharpuri (8.79% at 30X) made the most significant contributions in the genomic overlap of structural variants among the assessed breeds, reflecting their differential representation in the reference assembly. It is noteworthy that the genome coverage from other CNV studies in cattle (6%) and yak (5.7–6%) also falls within the same range [48–50]. Recently, Yang et al. [30] identified 21,152 CNVRs, representing 1.99% of the bubaline autosome length using multiple algorithms. Additionally, a couple of studies have discussed the chromosomal distribution of CNVRs including sex chromosomes, X and Y [51].

**Table 3:** Descriptive statistics revealing the average length and genomic coverage of CNVRs across different breeds

Breeds	Average length of CNVRs (bp)		Genomic Coverage (%)		Perfect match 10X_30X (%)
	10X	30X	10X	30X	
**Banni**	194877.20	6031.94	5.78	2.18	23.97
**Bhadawari**	203572.37	18790.23	5.96	6.08	31.60
**Pandharpuri**	201723.91	24411.12	5.88	8.79	29.31
**Murrah**	202088.91	6721.18	6.00	2.02	25.79
**Surti**	209642.68	7081.54	5.93	1.93	25.71
**Jaffarabadi**	197439.58	17838.28	5.94	6.53	31.50
**Average**	**201557.44**	**13479.05**	**5.92**	**4.59**	**27.98**

Remarkably, a higher level of sequence coverage or deeper sequencing was found to be beneficial for the precise detection of CNVs in buffaloes. This is in line with the previous research showing that greater sequencing coverage being linked to better sensitivity in CNV detection [52]. Prior studies have also suggested that coverage of 4X may be sufficient for detecting CNVs using the read depth method [53]. The average size of CNVRs scored in different breeds at 10X and 30X coverages was 0.201 Mb and 0.013 Mb, respectively. Table 4 depicts the distribution and relations of CNVRs in relation to gene density in different buffalo breeds across autosomes at twin coverages. Chr_13 was found to be gene-poor (in terms of density) but with the highest number of CNVRs (63–85 CNVRs at 10X coverage and 717–812 CNVRs at 30X coverage) in different breeds. Most of the clustering of CNVRs was evident in telomeric and sub-telomeric regions across different autosomes in most breeds. The least number of CNVRs were present on the smallest autosome (Chr_23) at both sequencing coverages, except Banni (10X) and Pandharpuri (30X).

**Table 4:** Distribution and relation of CNVRs with gene density in six buffalo breeds at two different densities across autosomes

Chr	Size (Mb)	Gene count	Gene density (genes /Mb)	CNVR count (10X)						CNVR count (30X)					
				BAN	PAN	SUR	JFR	MUR	BDW	BAN	PAN	SUR	JFR	MUR	BDW
**1**	202.35	1986	09.81	33	35	33	34	37	33	754	550	529	653	585	590
**2**	188.16	2760	14.67	35	36	38	40	41	39	631	628	484	632	563	532
**3**	174.87	3089	17.66	37	38	39	35	34	39	525	488	388	476	413	407
**4**	164.97	2505	15.18	48	44	37	51	49	39	631	527	446	598	506	499
**5**	132.50	2083	15.72	42	39	44	40	44	45	580	541	438	516	465	467
**6**	120.42	2126	17.65	29	21	24	20	22	25	414	333	311	370	331	301
**7**	117.00	1043	08.91	20	21	16	17	19	17	370	359	279	349	318	313
**8**	119.32	1351	11.32	23	29	25	22	25	23	417	333	309	381	338	357
**9**	110.26	2008	18.21	25	21	30	29	22	30	430	400	319	411	372	387
**10**	104.55	938	08.97	12	12	18	16	16	15	391	316	281	337	321	310
**11**	102.42	1697	16.57	27	34	27	25	34	34	370	319	275	350	323	310
**12**	106.39	1523	14.32	12	8	17	9	13	10	281	290	223	270	249	226
**13**	89.36	695	07.78	85	63	75	83	69	77	812	739	717	773	745	732
**14**	83.48	1293	15.49	11	9	5	10	10	12	246	226	195	230	186	189
**15**	81.83	856	10.46	11	5	9	7	7	10	216	208	165	224	204	195
**16**	85.12	1702	20.00	35	35	40	51	44	34	500	410	385	449	414	386
**17**	72.60	994	13.69	11	11	12	14	15	11	264	242	201	228	213	208
**18**	65.79	1874	28.48	28	28	27	33	29	34	339	315	253	319	273	275
**19**	71.58	591	08.26	8	10	9	9	7	10	257	218	204	228	229	202
**20**	69.61	1019	14.64	29	29	30	26	28	29	310	324	255	284	270	247
**21**	60.62	852	14.05	9	7	6	10	9	9	150	163	109	123	112	110
**22**	61.77	600	09.71	4	7	7	5	7	5	207	173	147	200	172	162
**23**	52.10	712	13.67	18	14	17	13	14	16	191	188	155	176	160	168
**24**	42.17	1048	24.85	6	3	2	1	6	4	116	170	82	107	98	94

Chr: Chromosome; Mb: Mega base; BAN: Banni; PAN: Pandharpuri; SUR: Surti; JFR: Jaffarabadi; MUR: Murrah; BDW: Bhadawari

**Fig. 3:**
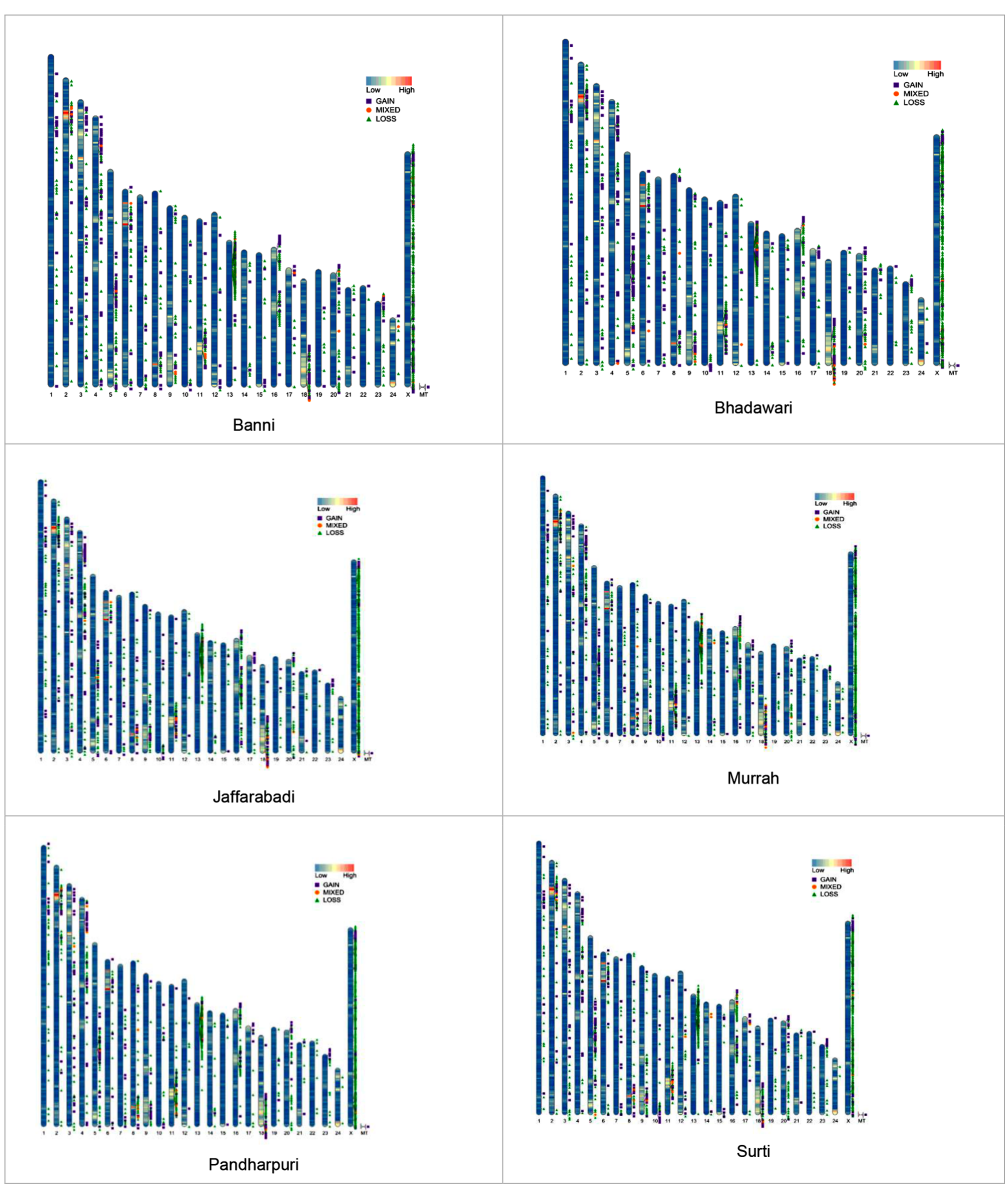
The ideograms depicting the distribution of CNVRs in different bubaline breeds at 10X WGS coverage

Overall, the distribution of CNVRs at both the coverages was uneven, which was expected given the functionality of corresponding genes or regulatory genetic elements. Figure 3 represents the distribution of CNVRs on different chromosomes of the bubaline genome in relation to the gene density, using ideograms, across different breeds at 10X coverage.

### Gene profiling

The longest CNVRs of each breed at both the coverages were evaluated, and the genes harbored on the top five CNVRs were shortlisted. The longest CNVR in Banni was located on autosome 16 at 10X, spanning 0.573 Mb. It harboured several genes such as *SRGAP2, IKBKE, RASSF5, ELF2D, DYRK3, MAPKAPK, IL-10, IL-19, IL-20* and *IL-24*. Interestingly, some of the genes overlapped with those observed in other breeds as well. For example, the *ZEB2* was shared among all the breeds, which is known to play a crucial role in the transforming growth factors β-signaling pathway, and its effects on growth, weight traits, and horn ontogenesis in cattle [54, 55]. *IL-10* gene is responsible for maintaining body homeostasis by resolving acute inflammation [56]. *IKBKE*, on the other hand, has been reported to regulate the maternal immune response during conceptus implantation in cattle [57]. Similarly, Oliveira et al. [58] reported the regulatory role of *SDC1* gene in controlling the milk yield in Ayrshire cattle. In addition, the largest CNVRs of Bhadawari encompassed different genes such as *KHDRBS2*, *DYNC2I1*, and *VIPR2*. Among these genes, *KHDRBS2* has been associated with reproductive traits in goats and Brahman cows, as well as adaptability in Colombian cattle [59]. In Jaffarabadi, the longest CNVR (0.573 Mb) was present on chromosome 16. The unique genes that showed overlap with the largest (top five) CNV regions were *LDAH*, *GDF7*, *HS1BP3*, *U4*, *OSR1*, and *7SK*. Studies have shown that *LDAH* promotes triglyceride production [60] while *GDF7* plays a role in seminal growth and neuronal development [61]. In Surti, the longest CNVRs harboured genes like *DYNC2I2*, *VIPR3*, *COA1*, *STK17A*, and *HECW1*, with *COA1* being implicated in mitochondrial translation thereby contributing to fitness and longevity. In mammals, the genomic region of *COA1* is recognized as a prominent evolutionary breakpoint area in which a combined deletion of *STK17A* and *COA1* genes was studied in rodents [62]. The largest CNVR in this breed was found on chromosome 2, which spanned a size of 0.668 Mb. Furthermore, *SFMBT2* gene was observed in Murrah, with the longest CNVR on chromosome 2 similar to Surti, covering a size of 1.17 Mb. This gene is important for trophoblast maintenance, placental development, and regulation of chondrocyte proliferation [63]. Finally, in Pandharpuri, the greatest CNVR spanned 0.729 Mb of buffalo genome with genomic coordinates spreading across chromosome 2. *PARD3* gene was identified in one of the top CNVRs, which is involved in cell growth and division as well as the formation of tight junctions in epithelial cells [64].

On the other hand, at 30X coverage, Banni showed the largest CNVR on chromosome 3, with a length of 0.337 Mb. The region contained several genes, including *TMEM45A, ZBTB12, EHMT2, SLC44A4, NEU1, SNORD52, SNORD48*, and two heat shock proteins namely *HSPA1A* and *HSPA1L*, which are involved in normal cell growth and survival, as well as protecting cytotoxic conditions [65]. *HSPA1A* and *HSPA1L* genes are reported to have sequence similarity arising from the duplication of genes offering thermotolerance at the cellular level [66]. Similarly, Bhadawari and Surti also showed large CNV regions on the same chromosome, with comparatively smaller sizes of around 0.285 Mb for both breeds. In Bhadawari, the genes such as *TRAPPC3, COL8A2, ADPRS, TEKT2*, and genes of the Argonaute family (*AGO1*, 3 and 4) showed overlap with CNVRs. The eukaryotic AGO proteins are active mediators in RNA silencing and other cellular processes [67]. For Surti, the genes exactly overlapped with that of the Bhadawari breed. Among the reported genes, Collagen VIII (*COL8*) is reported to play a major role in vascular integrity [68]. Jaffarabadi carried the longest CNVR on chromosome 14, which was the smallest one among all the breeds (0.246 Mb) contributing only 0.0089% of the genomic size. *RGS7* gene was observed in Jaffarabadi, belonging to the G-protein signaling family, that are significant in regulating a wide range of neuronal processes, such as vision, and nociception in mammalian species [69]. This gene was also identified as a candidate for milk production in Holstein cattle [70]. Considering the top five CNVRs of Murrah, the following genes were identified: *COA1, STK17A, RGS7, DYNC2I1, VIPR2*, and *HECW1*. Among these, *STK17A* gene is significantly involved in apoptosis, which has functions in immune response and disease resistance [71]. In contrast, *VIPR2* gene encodes a receptor that responds to vasoactive intestinal peptide (*VIP*), which helps with smooth muscle relaxation and the secretion of exocrine and endocrine glands. Mahoney et al. [72] also found that interfering with VIP production leads to a delay and reduction in the luteinizing hormone (LH) surge. Another gene, *HECW1*, is highly active in nerve cells and participates in the regulation of protein homeostasis, which has implications for both longevity and conditions related to ageing [73]. In sheep, studies have also reported this gene to be involved in variation with regard to the number of lambs born and have identified it as a target of selection in the Luzhong mutton sheep breed [74, 75]. Strikingly, the longest CNVR among all the breeds was present on chromosome 5 in Pandharpuri (0.475 Mb), covering 0.0171% of the genome. Consequently, this region showed overlap with multiple genes, including olfactory receptor genes (*OR10J5, OR10J1, OR10J4, and OR10J3), KANSL2, SNORA2C, LALBA, CRP, APCS, TUBA1B, LMBR1L, DHH, RHEBL1*, and *KMT2D*. Zhou et al. [76] described the potential role of olfactory genes in yak for the perception of chemical stimuli, which is very crucial for reproduction, acquiring basic needs such as food and mate, high altitude adaptation, and ultimately the survival of the animal. Another gene, Tubulin alpha 1b (*TUBA1B*) is an important component in the formation of the cytoskeleton, which is involved in immune cell infiltration, cell movement and within-cell transport [77]. Additionally, *LALBA* gene polymorphisms have been shown to influence milk production traits and somatic cell count in Polish Holstein-Friesian cows [78]. Interestingly, many of the genes identified at 30X in different breeds were similar to those scored at 10X coverage.

The genes overlapping the CNVs provided useful insights into the evolutionary history of these breeds. The genes present in all six populations were elucidated in DAVID. These genes were involved in the significant enrichment of pathways including oxidative phosphorylation, thermogenesis and pentose phosphate pathway. Significant enrichment was also noticed for biological processes like mitochondrial electron transport, regulation of presynapse assembly and actin filament organization. Similarly, cellular components including the respiratory chain, mitochondrial respiratory chain complex I and mitochondrial inner membrane were mainly involved while molecular functions like NADH dehydrogenase activity and metal ion binding were significantly enriched. The network of hub genes as identified using STRING-DB has been presented in supplementary Fig. 1.

## Conclusions

The present study provides new insights into the genetic variations among six important buffalo breeds. The findings from the present study elaborate on the evolutionary differences of six global buffalo breeds in terms of structural variants i.e., CNVs and CNVRs. Interestingly, a higher level of sequence coverage or deeper sequencing was found to be beneficial for the precise detection of CNVs in buffaloes with finer details. The genomic coverage of CNVs and CNVRs in these buffalo breeds varied amongst themselves. The highest genomic coverage of CNVRs was found for Murrah (6.00%) and Pandharpuri (8.79%) breeds. The results offered potential candidate genes linked to performance differences that could be used for selective breeding in the future. The CNV and CNVR maps for different buffalo breeds may be useful for the association of these structural variants with important (re)production and adaptability traits.

## Methods

### Sampling and whole genome resequencing data

The present study was undertaken on whole genome resequencing data on 75 buffaloes (*Bubalus bubalis*) representing six distinct breeds, with 12 animals each from Murrah, Surti, and Banni and 13 animals each from Bhadawari, Jaffarabadi, and Pandharpuri populations (Table 5). These breeds have been imported by different nations courtesy their high genetic merit and proven performance with respect to economic traits and have been integrated into the breeding policy for improvement of buffaloes in these countries [5]. The sequencing data was retrieved from an online database and was based on an earlier publication report [79]. The samples were obtained from breeding tracts of respective buffalo breeds (as given in Table 5) and sequenced using two different platforms as described by Dutta et al. [79]. Briefly, paired-end sequencing data were generated at two sequencing centres using different coverages and sequence read lengths. One set of samples was sequenced using NEBNext Ultra DNA Library Prep Kit (library preparation) and Illumina HiSeq 2500 sequencing platform at SciGenom Labs (India) at 10X coverage with a read length of 250 bp. Whereas, the rest of the samples were sequenced using Illumina TruSeq Nano DNA Library Prep Kit (library preparation) and Illumina HiSeq X sequencing platform at Edinburgh Genomics (United Kingdom) at 30X coverage with a read length of 150 bp. The average sequencing coverage for samples at two centres (SciGenom Labs, India, and Edinburgh Genomics, United Kingdom) was 8X and 37X, respectively. In nutshell, whole genome resequencing was retrieved on these animals across two different coverages: 10X for six animals from each breed and 30X for the rest, as indicated in Table 5.

**Table 5:** Sample size and details of buffalo breeds used for the elucidation of copy number variations using whole genome resequencing data

Breeds	Major breeding tract	Sample size		
		10X coverage	30X coverage	Total
**Murrah**	Haryana	6	6	12
**Bhadawari**	Uttar Pradesh and Madhya Pradesh	6	7	13
**Jaffarabadi**	Gujarat	6	7	13
**Pandarpuri**	Maharashtra	6	7	13
**Surti**	Gujarat	6	6	12
**Banni**	Gujarat	6	6	12
**Total**		**36**	**39**	**75**

The quality of the sequence reads was assessed with FastQC v0.12.1 [80] and poor-quality reads and adapters were removed via TrimGalore v0.6.5 [81] using default parameters. After trimming and reassessing the read quality, the Burrows-Wheeler aligner v0.7.12 [82] was used to index the genome NDDB_SH_1 of water buffaloes (release date: September 2021), which has a total sequence length of 2,622,460,639 bp [83]. The sequence reads, after quality control were mapped against the genome index using BWA-MEM algorithm with default settings [82].

### Post-alignment processing

Following alignment to the genome assembly, the individual sequence alignment map (SAM) files were converted into a more efficient binary alignment map format (BAM) using SAMtools v1.17 [84]. The output files were validated by Picard tools v2.25.1 [85] build under GATK v4.0.2.0 and sorted in concordance with genomic coordinates. Subsequently, the SM read tags were added to the mapped reads. Furthermore, the PCR duplicates in BAM files were marked for removal using the ‘MarkDuplicates’ function of Picard tools. This effectively minimizes the PCR amplification bias by clipping on 5′ read ends.

### CNV detection, filtering and concatenation

CNV detection and analysis were undertaken using read-depth-based software CNVnator v.0.4.1 [17]. The core principle behind CNVnator involves dividing the genome into non-overlapping bins set by the user and calculating the RD signal by counting the mapped reads within each bin. Following this, CNVnator performs statistical significance tests for CNV predictions [17]. In the present study, copy number histograms were generated from PCR duplicate-free Picard-BAM files for partitioning the CNV calls and for downstream statistical analysis. Subsequently, the optimal bin size for read depth analysis was chosen as the multiple of 100s in all the samples by considering the read depth, read length, distribution, and quality. The optimal bin size was selected based on the ratio of the read depth and its variance; fitting the recommended ratio between 4 and 5. Correction for GC waves was done within CNVnator, which is necessary to eliminate the GC bias resulting from reduced depth coverage at GC-rich regions [86, 87]. Subsequently, the CNV variants were individually called using the ‘call’ command in CNVnator.

After CNV detection, the quality control and post-pruning were done based on p-value, zero mapping quality (q0), and CNV size. The study considered the following parameters for filtering of CNVs: p-value calculated by t-test statistics < 0.01, variants with mapping quality < 0.5, and the size of CNVs < 1 kb and > 5 Mb, as suggested by previous studies [88]. The q0 filter (fraction of mapped reads with zero quality) of 0.5 was used in the present study that was indicative of the degree of certainty that a read comes from the location to which it is aligned.

A customized Python script was used to concatenate the overlapping CNVs (with minimum of one bp overlap) into copy number variation regions (CNVRs). The merged CNVRs were marked as deletion, duplication, or mixed depending on whether the events in proximal CNVs were all deletion, all duplication, or a mix of deletion and duplication. The whole analysis was done using the high-performance computing facility of Indiana University and Purdue University, Indianapolis (IUPUI), USA (now Indiana University Indianapolis).

The impact of sequence coverage on CNV detection was also investigated in study animals with two levels of sequence coverage (10X and 30X). Changes in the CNV count were attributable to the changes in sequencing coverage and read length. RIdeogram package [89] was used to plot the distribution of CNVRs in relation to the gene density. Briefly, the gene density parameters were elucidated from *gff* file corresponding to the genome assembly used in the study using a window of 1 Mb. The chromosomal coordinates were retrieved from assembly metadata. RIdeogram package was used to plot the CNVR data overlaid by the gene density parameters.

### Unique and overlapping CNVs and CNVRs

The chromosomal coordinates of structural variants from different populations were used in BEDTools v2.26.0 [90] to elucidate the common (with the same chromosomal coordinates), overlapping (with at least one bp overlap) and unique CNVs and CNVRs. Furthermore, the genomic coverage of CNVRs against the reference genome assembly was elucidated in each population.

### Gene profiling

The information on genes and genetic variants overlapping CNVRs in different breeds was extracted by mining data corresponding to their genomic coordinates from the genome annotation file (gtf file, corresponding to the NDDB_SH_1 genome assembly) using BEDTools program. The common genes found to overlap CNVRs in all six populations were used for profiling and functional annotation in Database for Annotation, Visualization and Integrated Discovery (DAVID). Furthermore, the longest CNVRs in terms of chromosomal coordinates in each breed were identified and overlapping genes were elucidated in Ensembl. The information on the functioning of these genes was retrieved by mining the information from PubMed and other relevant databases. Additionally, the common genes that showed overlap with CNVRs were processed in STRING-DB for identification of hub genes that are involved in various pathways in buffaloes.

### Electronic supplementary material

Below is the link to the electronic supplementary material.

Supplementary Material 1

## Data Availability

The original datasets supporting the conclusions of this article are available at (https://www.ebi.ac.uk/ena/browser/view/PRJEB39591) with accession number PRJEB39591.

## Abbreviations

BAM: Binary alignment map
CNV: Copy number variation
CNVR: Copy number variation region
DAVID: Database for Annotation, Visualization and Integrated Discovery
GDP: Gross domestic product
LH: Luteinizing hormone
MSB: Mean shift based
SAM: Sequence alignment map
SNP: Single nucleotide polymorphism
WGS: Whole genome (re)sequencing
