# Supplementary material for: Read-depth based approach on whole genome resequencing data reveals important insights into the copy number variation (CNV) map of major global buffalo breeds
Source: BMC Genomics. 2023 Oct 16;24:616. doi: 10.1186/s12864-023-09720-8 (PMC10580622; doi:10.1186/s12864-023-09720-8)
Supplement: Supplementary file 1 — Supplementary Material 1 [file 12864_2023_9720_MOESM1_ESM.docx]

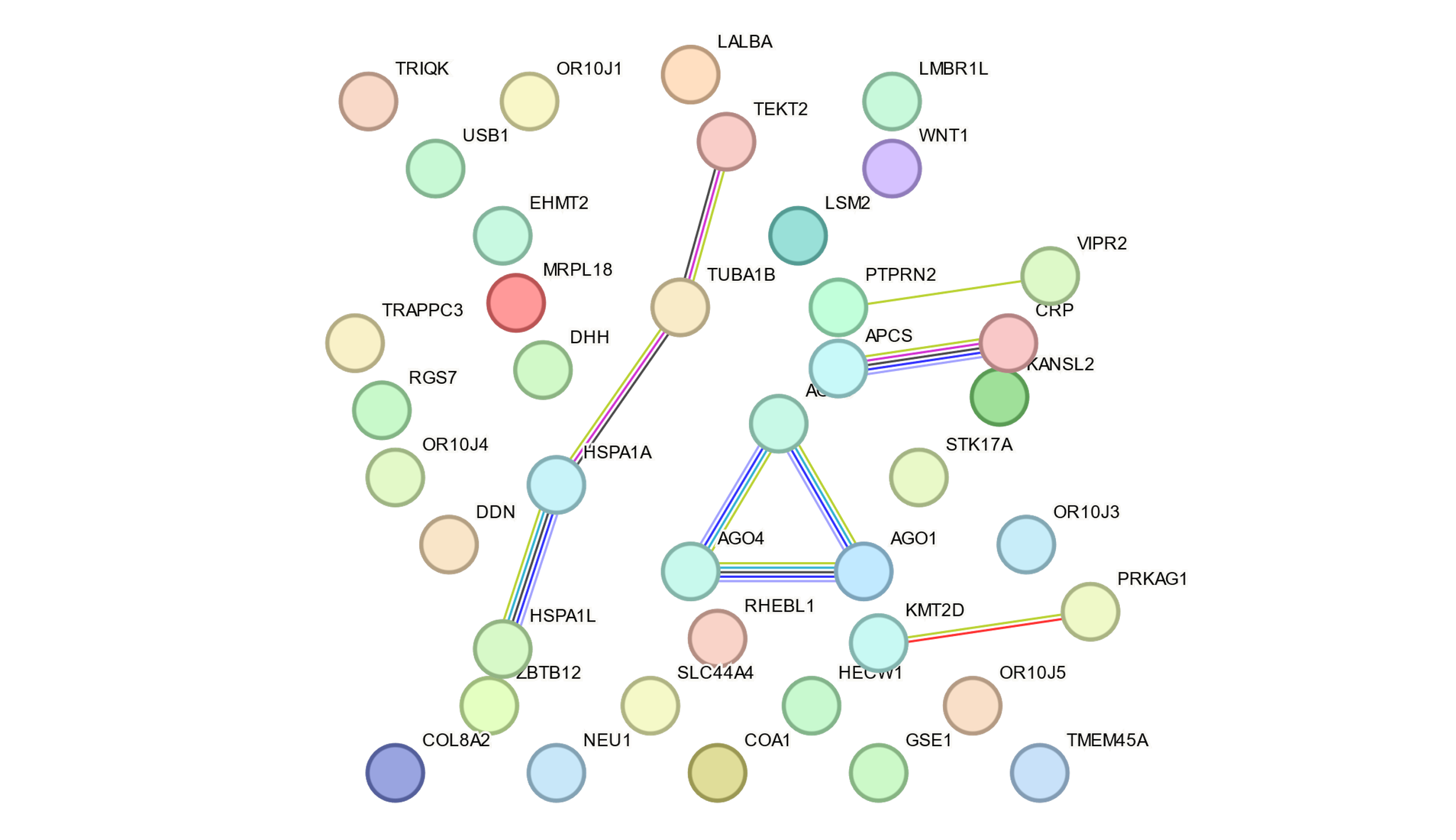
Supplementary Fig. 1: The gene-gene interaction network identifying the hub genes as identified using STING-DB
